# Cut-off Values of Body Mass Index, Waist Circumference, and Waist-to-Height Ratio to Identify Excess Abdominal Fat: Population-Based Screening of Japanese Schoolchildren

**DOI:** 10.2188/jea.JE20100116

**Published:** 2011-05-05

**Authors:** Yuki Fujita, Katsuyasu Kouda, Harunobu Nakamura, Masayuki Iki

**Affiliations:** 1 Department of Public Health, Kinki University Faculty of Medicine, Osakasayama, Japan; 2 Department of Health Promotion and Education, Graduate School of Human Development and Environment, Kobe University, Kobe, Japan

**Keywords:** child, screening, obesity, statistics as topic, reference values

## Abstract

**Background:**

School-based screening and prevention programs for adiposity generally target school children in grades 4 and 6 (age 9–11 years). The aims of this study were to evaluate the validity of body mass index (BMI), waist circumference (WC), and waist-to-height ratio (WHtR) in identifying abdominal adiposity in fifth-grade Japanese school children and to determine optimal cut-off values for anthropometric measures.

**Methods:**

The target population was fifth-grade school children enrolled in 2 schools in Shizuoka, Japan between 2008 and 2010; 422 of the 466 children participated in the present study. Abdominal adiposity was defined as percent trunk fat in the 95th percentile or higher, as determined by dual-energy x-ray absorptiometry (DXA). We analyzed the validity of BMI, WC, and WHtR using receiver operating characteristic (ROC) curve analysis. The Youden index was used to determine cut-off values of BMI, WC, and WHtR that identify excess abdominal fat.

**Results:**

Optimal cut-off values to identify abdominal adiposity were 20.8 kg/m^2^ (BMI), 76.5 cm (WC), and 0.519 (WHtR) for boys, and 19.6 kg/m^2^ (BMI), 73.0 cm (WC), and 0.499 (WHtR) for girls. Areas under the ROC curve were 0.983 (BMI), 0.987 (WC), and 0.981 (WHtR) for boys, and 0.981 (BMI), 0986 (WC), and 0.992 (WHtR) for girls.

**Conclusions:**

BMI, WC, and WHtR successfully identified a high proportion of children with excess abdominal fat as measured by DXA, demonstrating that these measures are useful indices for school screening.

## INTRODUCTION

Obesity is defined as the presence of excess body fat. A cohort study demonstrated an association between obesity in childhood and heart disease in adulthood.^1^ Recent studies have also reported that abdominal adiposity in children is associated with risk factors for cardiovascular and metabolic disease.^2^^,^^3^ Therefore, school-based screening is conducted in many countries to prevent obesity and abdominal adiposity.^4^^–^^8^ These screening and intervention programs generally target school children in grades 4 and 6 (age 9–11 years).^9^^–^^15^

Body mass index (BMI) was reported to be closely associated with abdominal adiposity in Sweden.^16^ A relationship between waist-to-height ratio (WHtR) and abdominal fat was observed in an Australian health and fitness survey.^17^ In addition, waist circumference (WC) was reported to be an index of trunk fat in white children.^18^ Accordingly, BMI, WHtR, and WC are recommended as screening tools to identify abdominal adiposity. However, no population-based studies have evaluated the validity of BMI, WC, and WHtR as indicators of abdominal adiposity in Japanese children. The aim of this study was to assess the validity of these anthropometric indices in fifth-grade Japanese school children. We also determined the optimal cut-off values of BMI, WC, and WHtR for the identification of excess abdominal fat, as measured by dual-energy x-ray absorptiometry (DXA), which is the standard technique for measuring fat mass.

## METHODS

### Study population

The target population was fifth-grade school children (age 10 years) in Shizuoka, Japan who attended Aritama Elementary School between 2008 and 2010 or Fukuroi-kita Elementary School in 2009. We excluded 31 subjects for whom we could not obtain informed consent from both child and a parent and 13 subjects who were absent from school on the day of the examination. Of the 466 children (252 boys, 214 girls) enrolled in these schools, 422 (226 boys, 89.7%; 196 girls, 91.6%) participated in this study.

### Ethical considerations

Informed written consent was obtained from each child and a parent. This study was approved by the Ethics Committee of Kinki University Faculty of Medicine and was conducted in accordance with the guidelines of the Declaration of Helsinki.

### Measurements

BMI was calculated as weight divided by height squared (kg/m^2^). We measured WC using the method recommended by the Ministry of Health, Labour and Welfare.^19^ WC was measured to the nearest centimeter with a non-elastic flexible tape at the umbilicus level while the subject was standing, keeping the tape measure horizontal around the body. Measurements were taken at the end of normal expiration, not during breath-holding or abdominal muscle contraction. When the umbilicus was pulled downward by fat accumulation, WC was measured midway between the anterior superior iliac spine and the lowest portion of the rib cage. WHtR was calculated as WC divided by height.

Whole-body and regional (arms, legs, and trunk) body fat of all children were determined with a single DXA scanner that was brought to the schools in a mobile test room. Measurements of both arms, both legs, and the head were isolated from trunk measurements using computer-generated default lines with manual adjustment in the anterior view planogram. Specific anatomical landmarks (chin, center of the glenohumeral joint, and femoral neck axis) defined the trunk. Total and regional fat levels were calculated as percentage of total body mass [fat mass/(fat mass + lean tissue mass + bone mineral content)] × 100%. We selected the 85th, 90th, and 95th percentile values to define the lower limits of excess abdominal fat and abdominal adiposity according to sex.

### Statistical analysis

Data were analyzed with SAS software for Windows version 9.1 (SAS Institute Japan Ltd., Tokyo, Japan). The overall significance level was set at α = 0.05. Height, weight, BMI, WC, and WHtR were expressed as mean ± standard deviation (SD), and body fat (%), trunk fat (%), upper limb (%), and lower limb (%) values represented geometric means with values for mean − SD and mean + SD in parentheses (Table 1). To determine the necessity of analysis by sex, the mean anthropometric indices of boys and girls were compared by Student *t*-test.

**Table 1.** Participant characteristics

Variable	Boys (*n* = 226)				Girls (*n* = 196)				*P*
	
	Mean ± SD	SE	Min	Max	Mean ± SD	SE	Min	Max	
Height (cm)	141.9 ± 6.5	0.4	124.7	165.0	144.2 ± 6.2	0.5	129.2	157.9	<0.01
Weight (kg)	34.6 ± 6.8	0.5	22.4	59.9	35.2 ± 6.9	0.5	21.5	77.1	0.34
BMI (kg/m^2^)	17.1 ± 2.3	0.2	13.3	26.0	16.8 ± 2.4	0.2	12.7	31.3	0.32
WC (cm)	63.8 ± 6.9	0.5	50.5	87.0	63.7 ± 6.6	0.5	52.5	101.0	0.87
WHtR	0.45 ± 0.04	0.003	0.39	0.59	0.44 ± 0.04	0.003	0.37	0.64	0.05
Body fat (%)	18.0 (13.2, 24.6)	1.02	9.2	39.1	19.8 (15.5, 25.4)	1.02	11.0	39.9	<0.01
Trunk fat (%)	5.1 (3.2, 8.0)	1.03	2.1	16.8	5.8 (3.9, 8.5)	1.03	2.5	17.7	<0.01
Upper limb (%)	2.2 (1.5, 3.2)	1.03	1.0	5.2	2.4 (1.7, 3.2)	1.02	1.2	5.7	0.07
Lower limb (%)	8.2 (6.0, 11.2)	1.02	3.5	15.6	9.3 (7.3, 11.9)	1.02	4.6	15.6	<0.01

Abbreviations: BMI, body mass index; WC, waist circumference; WHtR, waist-to-height ratio.

Body fat (%), trunk fat (%), upper limb (%), and lower limb (%) values represent geometric means, with values for M − SD and M + SD in parentheses.

The sensitivity and specificity of BMI, WC, and WHtR as indicators of abdominal adiposity were determined with cut-off values. Receiver operating characteristic (ROC) curves and area under the curve (AUC) for ROCs were obtained by plotting sensitivity against the false-positive rate (1 − specificity). The Youden index (J) was used to determine optimal cut-off values of BMI, WC, and WHtR for identification of excess abdominal fat (J = sensitivity + specificity − 1).

## RESULTS

Characteristics of the study participants are shown in Table 1. Mean BMI and WC values did not differ significantly according to sex; however, WHtR was slightly higher for boys than for girls (*P* = 0.05). Percentages of body fat, trunk fat, and lower limb fat were significantly higher for girls than for boys, but upper limb fat percentage did not differ significantly by sex.

The Figure shows ROC curves for BMI, WC, and WHtR as indicators of excess abdominal fat. As shown in Table 2, AUCs were ≥0.98 for BMI, WC, and WHtR as indicators of excess abdominal fat (≥95th percentile) for both sexes. The AUC value for BMI in boys was similar to that for WC and WHtR. For girls, the AUC value for WHtR was slightly higher than the other anthropometric measures.

**Figure.**
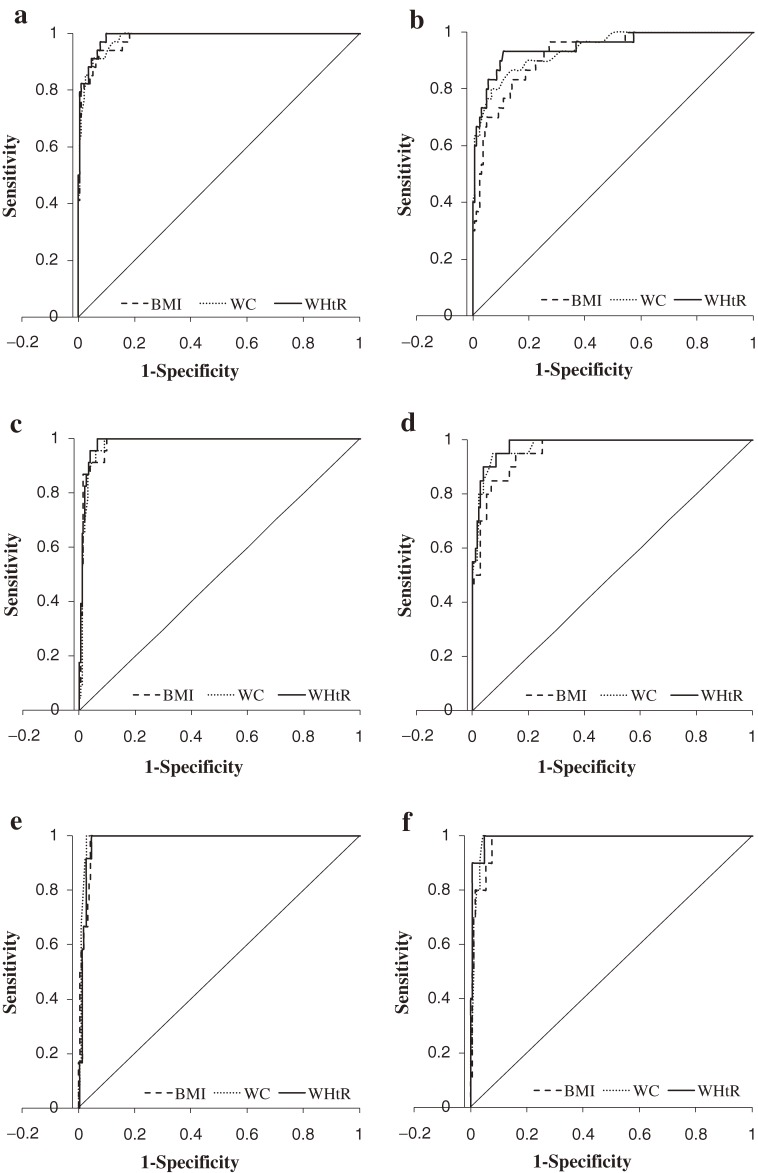
Area under receiver operating characteristic curve (AUC) for anthropometric variables as indicators of abdominal adiposity. Body mass index (BMI), waist circumference (WC), and waist-to-height ratio (WHtR) were evaluated for the ability to identify excess abdominal fat in fifth-grade school children. Excess abdominal fat was defined as percent abdominal fat greater than the 85th percentile value for (a) boys and (b) girls, greater than the 90th percentile value for (c) boys and (d) girls, or greater than the 95th percentile value for (e) boys and (f) girls.

**Table 2.** Area under the receiver operating characteristic curves for BMI, WC, and WHtR as indicators of excess trunk fat in fifth-grade Japanese school children

Trunk fat (%)	Measure	Boys		Girls	
	
		AUC	95% CI	AUC	95% CI
85th	BMI	0.980	0.964–0.997	0.924	0.877–0.971
percentile	WC	0.980	0.964–0.996	0.932	0.882–0.982
	WHtR	0.987	0.976–0.999	0.951	0.905–0.995

90th	BMI	0.982	0.966–0.998	0.959	0.927–0.991
percentile	WC	0.980	0.964–0.997	0.976	0.952–1.000
	WHtR	0.986	0.973–0.999	0.981	0.963–0.998

95th	BMI	0.983	0.968–0.999	0.981	0.961–1.001
percentile	WC	0.987	0.974–1.001	0.986	0.971–1.001
	WHtR	0.981	0.964–0.998	0.992	0.981–1.004

Abbreviations: AUC, area under curve; BMI, body mass index; CI, confidence interval; WC, waist circumference; WHtR, waist-to-height ratio.

Optimal cut-off values of BMI, WC, and WHtR for identification of excess abdominal fat are shown in Table 3. The cut-off values to identify abdominal adiposity for boys were higher than those for girls: BMI, 20.8 kg/m^2^ vs 19.6 kg/m^2^; WC, 76.5 cm vs 73.0 cm; and WHtR, 0.519 vs 0.499, respectively. The sensitivity and specificity of BMI, WC, and WHtR as indicators of excess abdominal fat were high for both sexes.

**Table 3.** Optimal cut-off values of BMI, WC, and WHtR as indicators of excess trunk fat in fifth-grade Japanese school children

Trunk fat (%)	Measure	Boys			Girls		
	
		Cut-off	Sensitivity	Specificity	Cut-off	Sensitivity	Specificity
85th percentile	BMI	18.6	0.94	0.93	17.0	0.97	0.73
	WC	68.5	0.91	0.95	68.0	0.80	0.93
	WHtR	0.467	1.00	0.90	0.460	0.93	0.89

90th percentile	BMI	18.7	1.00	0.90	17.8	0.95	0.85
	WC	68.5	1.00	0.91	69.0	0.95	0.93
	WHtR	0.488	1.00	0.94	0.460	1.00	0.87

95th percentile	BMI	20.8	1.00	0.96	19.6	1.00	0.92
	WC	76.5	1.00	0.97	73.0	1.00	0.96
	WHtR	0.519	1.00	0.95	0.499	1.00	0.95

Abbreviations: BMI, body mass index; WC, waist circumference; WHtR, waist-to-height ratio.

## DISCUSSION

This is the first report to demonstrate the validity of BMI, WC, and WHtR as identifiers of abdominal adiposity for population-based screening in Japanese school children. The high AUCs obtained by ROC curve analysis indicated the high validity of these anthropometric measures in distinguishing excess fat from a normal level of fat, as determined by DXA. Optimal cut-off values for BMI were 20 kg/m^2^ for boys (AUC: 0.983, sensitivity: 1.00, specificity: 0.90) and 19 kg/m^2^ for girls (AUC: 0.981, sensitivity: 1.00, specificity: 0.90), optimal cut-off values for WC were 76 cm for boys (AUC: 0.983, sensitivity: 1.00, specificity: 0.89) and 73 cm for girls (AUC: 0.981, sensitivity: 1.00, specificity: 0.96), and optimal cut-off values for WHtR were 0.50 for boys (AUC: 0.983, sensitivity: 1.00, specificity: 0.93) for boys and 0.49 for girls (AUC: 0.981, sensitivity: 1.00, specificity: 0.95).

BMI was previously reported to be strongly associated with abdominal fat.^16^^,^^20^^,^^21^ Dencker et al also reported that BMI was strongly correlated with total body fat and abdominal fat mass for boys (*r* = 0.94 and *r* = 0.93, respectively) and girls (*r* = 0.95 and *r* = 0.95, respectively) in Sweden.^16^ Similarly, Pietrobelli et al reported that BMI was strongly associated with total body fat in healthy Italian children (*R*^2^ = 0.85, boys; *R*^2^ = 0.89, girls).^21^ The relationship between BMI and abdominal adiposity observed in the present study is consistent with that reported in previous studies.

The BMI cut-off values for abdominal adiposity obtained in the present study were similar to those reported by the International Obesity Task Force (IOTF). The IOTF constructed percentile curves for BMI using the LMS (lambda, mu, sigma) method and reported that the BMIs of 10.5-year-old boys and girls corresponding to a BMI of 25 kg/m^2^ at age 18 years were 20.2 and 20.3, respectively.^22^

Diagnostic criteria for metabolic syndrome in adults have been used for specific health checkups and counseling in Japan.^23^ However, the suggested cut-off values for obesity and abdominal adiposity are controversial.^24^ Furthermore, another method has been developed to identify excess abdominal fat.^25^ For children, the cut-off values recommended by a research group of the Japanese Ministry of Health, Labour and Welfare were 75 cm for WC and 0.5 for WHtR.^26^ However, in healthy Japanese children, the validity of these cut-off points is unclear, as are the diagnostic criteria for metabolic syndrome in adults. In the present study, WC was a highly accurate indicator of excess abdominal fat in Japanese school children, as demonstrated by an AUC ≥0.98 for both sexes. This finding was consistent with a previous study reporting that WC successfully identified a high proportion of white children and adolescents with high percentages of trunk fat as measured by DXA (AUC = 0.97, sensitivity = 89%, specificity = 94%).^18^ We determined that the optimal cut-off values of WC to identify abdominal adiposity were 76.5 cm for boys and 73.0 cm for girls, which are similar to those recommended by the Japanese Ministry of Health, Labour and Welfare.^26^ However, the optimal WC cut-off value may vary according to age; therefore, additional studies assessing a wide age range are needed.

In the present study, analysis of WHtR as an indicator of abdominal adiposity resulted in high AUCs (0.98) for both sexes. We determined that the optimal cut-off values of WHtR to identify abdominal adiposity were 0.51 for boys and 0.50 for girls. The study group of the Japanese Ministry of Health, Labour and Welfare recommend a cut-off value of WHtR of 0.50,^26^ which is consistent with our findings.

The strength of this study is that it was population-based. Because DXA equipment is generally located in medical centers, it is difficult to obtain population-based data by DXA. Therefore, we transported DXA equipment to the schools. A limitation of this study is that data were not obtained from a group of children with a wide age range. However, data from fifth-grade school children are useful for school-based screening and intervention programs for cardiovascular disease,^4^^–^^8^ which generally target school children in grades 4 and 6.^9^^–^^15^ In addition, the study population came from only 2 schools in Shizuoka, Japan. To develop national criteria, additional studies in several other regions of Japan are needed. Finally, we did not evaluate the validity of the method used to measure WC; however, we used the method recommended by the Ministry of Health, Labour and Welfare.^19^

In conclusion, we demonstrated that BMI, WC, and WHtR are highly accurate indicators of excess abdominal fat in Japanese school children. Values obtained in the present population-based study were consistent with WC and WHtR cut-off values recommended by the Japanese Ministry of Health, Labour and Welfare. Our findings suggest that these anthropometric measures are useful indices for school screening and that WHtR is particularly useful because it is not dependent on age or sex and is easier to use.
